# Supplementary material for: Construction of a Novel Multigene Panel Potently Predicting Poor Prognosis in Patients with Clear Cell Renal Cell Carcinoma
Source: Cancers (Basel). 2020 Nov 22;12(11):3471. doi: 10.3390/cancers12113471 (PMC7700485; doi:10.3390/cancers12113471)
Supplement: Supplementary file 1 [file cancers-12-03471-s001.pdf]

Supplementary Materials: Construction of a Novel Multigene Panel Potently Predicting Poor Prognosis in Patients with Clear Cell Renal Cell Carcinoma

Xiaozeng Lin, Anil Kapoor, Yan Gu, Mathilda Jing Chow, Jingyi Peng, Pierre Major and Damu Tang

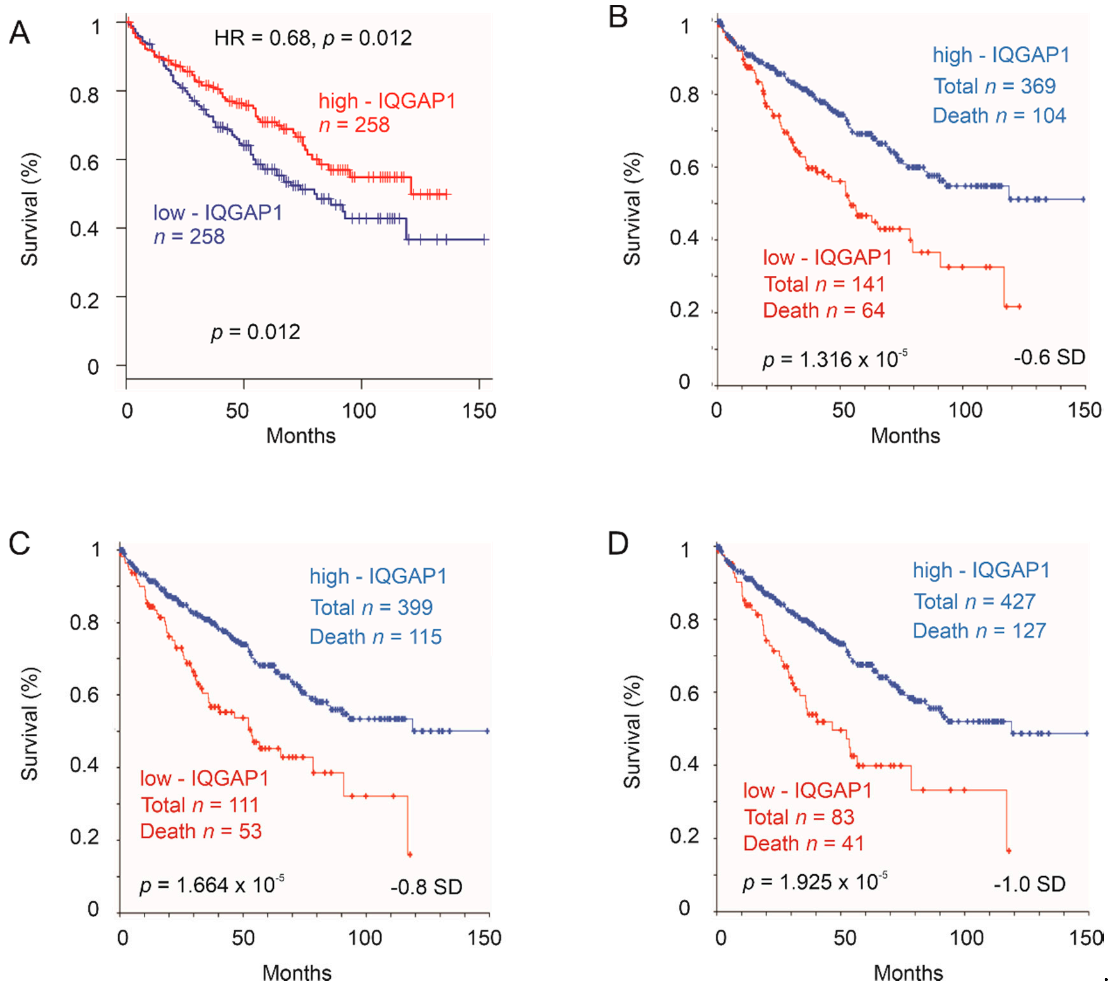

Figure S1. Downregulation of IQGAP1 is associated with reductions in overall survival in ccRCC. (**A**) Kaplan-Meier survival curve was constructed using the GEPIA2 database (http://gepia2.cancer-pku.cn/#index). (**B**–**D**) The TCGA PanCancer Atlas ccRCC dataset was divided into a high and low fatality risk group using the cutoff points of -0.6 SD, -0.8 SD, and -1 SD as indicated; the associated Kaplan-Meier survival curves were produced using tools provided by cBioPortal. Statistical analyses were carried out with logrank test.

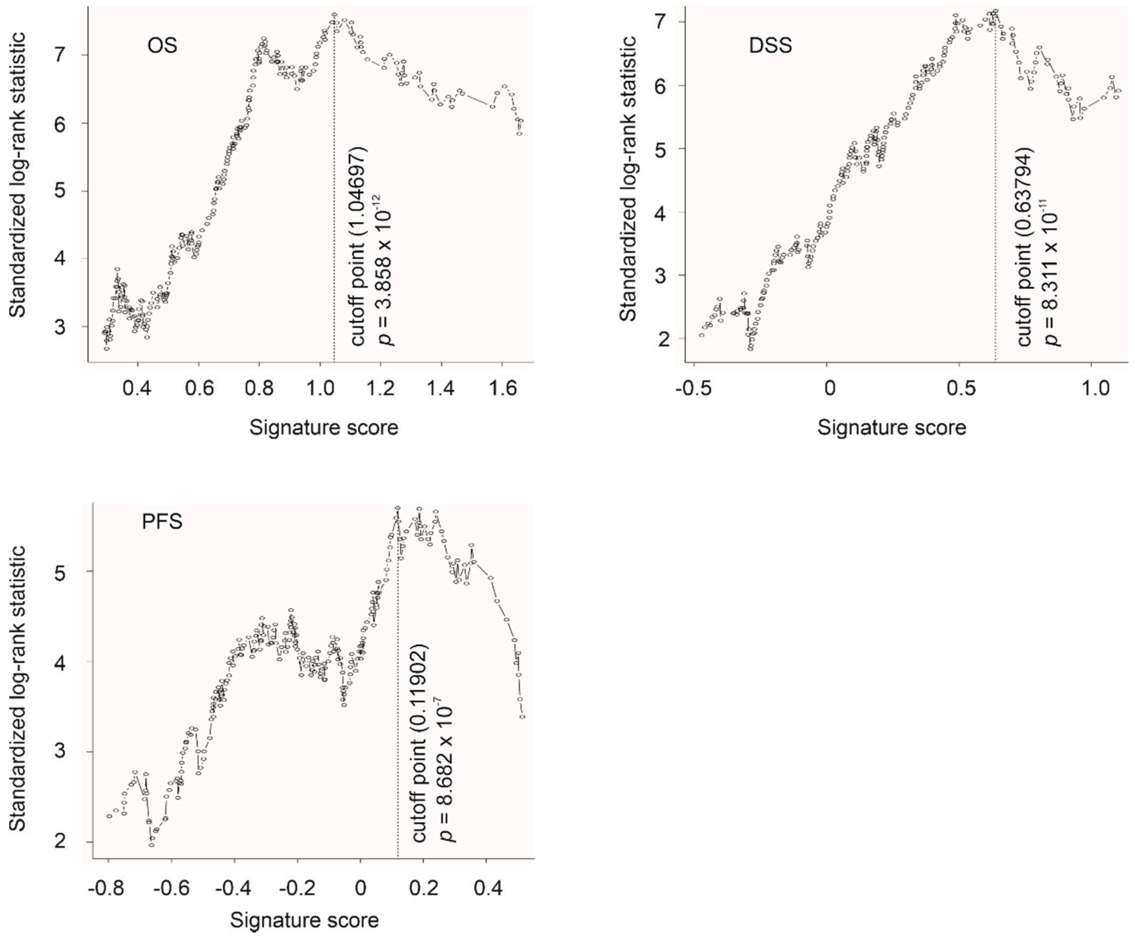

Figure S2. Cutoff points estimation for overall survival (OS), disease specific survival (DSS), and progression free survival (PFS). The respective cutoff points of SigIQGAP1NW scores were determined using Maximally Selected Rank Statistics (the Maxstat package) in R. Individual cutoff points and their associated *p* values are indicated.

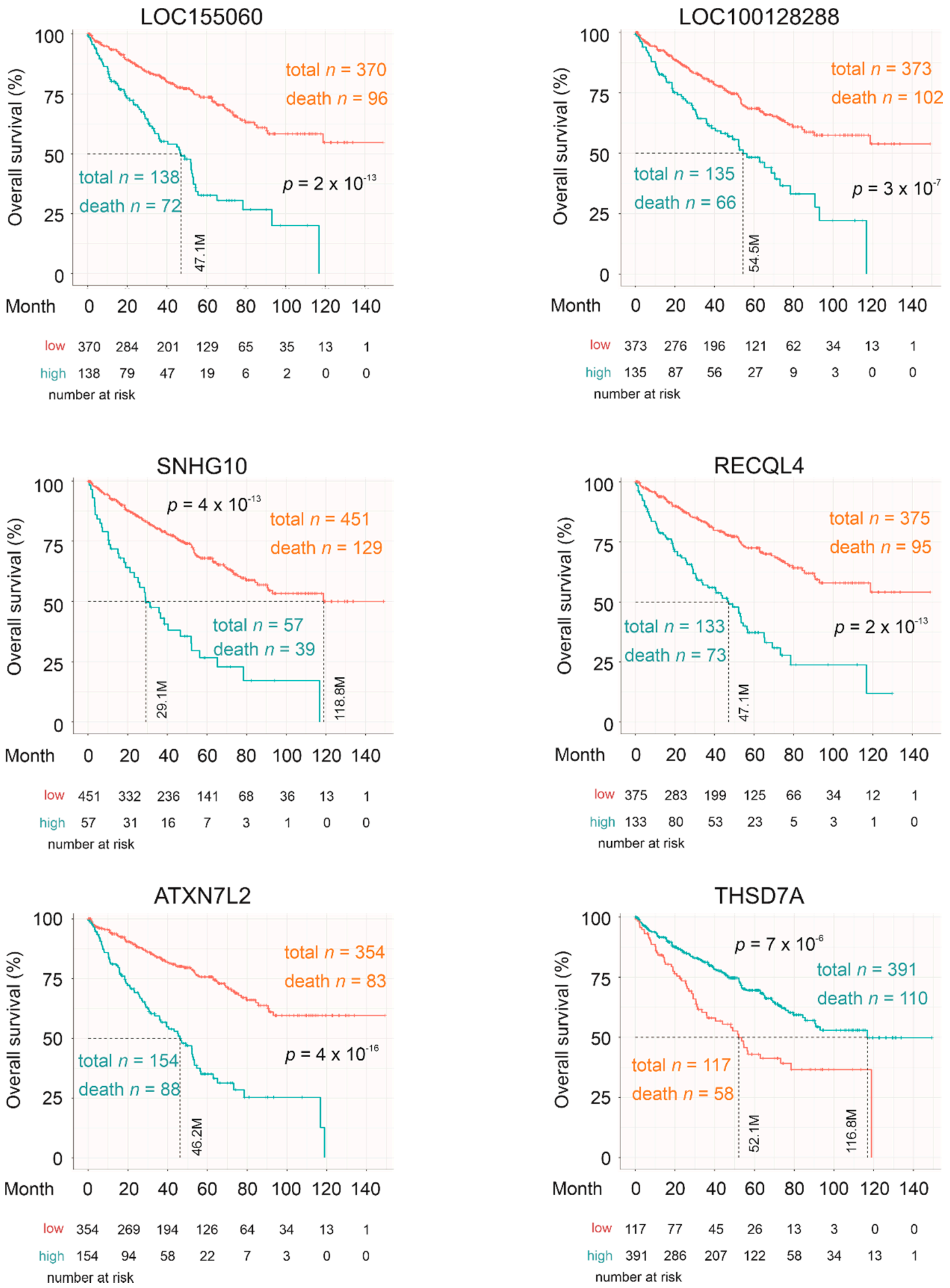

Figure S3. Kaplan-Meier survival curves for the indicated component genes of SigIQGAP1NW. Cutoff points for these component genes were determined based on their mRNA expression using Maximally Selected Rank Statistics (the Maxstat package) in R. The individual survival curves were produced using the R survival package. Statistical analyses were performed with logrank test.

Table S1. Differentially expression genes in ccRCCs with and without IQGAP1 downregulation.

Gene	Gene ID	Cytoband	Altered Group (Mean Log2)	Unaltered Group (Mean Log2)	Altered Group (SD)	Unaltered Group (SD)	Log2 Ratio	*p*-Value	q-Value
IQGAP1	8826	15q26.1	11.82	12.77	0.34	0.32	−0.95	1.96×10^{-61}	2.54×10^{-57}
OXLD1	339229	17q25.3	8.84	7.75	0.59	0.62	1.09	1.54×10^{-39}	6.66×10^{-36}
RC3H2	54542	9q33.2	7.44	8.81	0.78	0.61	−1.37	7.39×10^{-37}	1.59×10^{-33}
GLI4	2738	8q24.3	7.91	6.46	0.84	0.95	1.45	3.24×10^{-36}	5.25×10^{-33}
LNPEP	4012	5q15	6.95	8.57	0.95	0.84	−1.62	5.52×10^{-36}	7.15×10^{-33}
SLFN5	162394	17q12	8.02	9.54	0.89	0.68	−1.52	1.69×10^{-35}	1.68×10^{-32}
NBEAL1	65065	2q33.2	6.23	8.02	1.07	0.96	−1.79	3.38×10^{-35}	2.73×10^{-32}
TAOK1	57551	17q11.2	6.68	8.69	1.2	0.86	−2.01	5.28×10^{-35}	3.79×10^{-32}
PPP1R35	221908	7q22.1	8.48	7.22	0.76	0.74	1.26	1.04×10^{-34}	6.40×10^{-32}
MRPS26	64949	20p13	10.08	9.07	0.61	0.56	1	1.43×10^{-34}	8.43×10^{-32}
CCDC24	149473	1p34.1	8.05	6.75	0.78	0.84	1.3	2.17×10^{-34}	1.22×10^{-31}
PIK3CA	5290	3q26.32	7.95	9.06	0.67	0.47	−1.11	2.49×10^{-34}	1.35×10^{-31}
GTF3C4	9329	9q34.13	7.23	8.44	0.74	0.54	−1.22	6.57×10^{-34}	3.27×10^{-31}
FASTK	10922	7q36.1	10.79	9.83	0.59	0.62	0.96	7.54×10^{-34}	3.62×10^{-31}
UBXN7	26043	3q29	6.99	8.38	0.85	0.66	−1.39	9.19×10^{-34}	4.17×10^{-31}
CLOCK	9575	4q12	6.9	8.52	0.99	0.72	−1.62	9.35×10^{-34}	4.17×10^{-31}
KIAA0754	643314	1p34.3	5.62	7.93	1.43	1.12	−2.32	1.16×10^{-33}	4.99×10^{-31}
PTPN14	5784	1q32.3–q41	7.09	8.37	0.79	0.81	−1.28	1.48×10^{-33}	6.17×10^{-31}
SAMHD1	25939	20q11.23	8.7	10.23	0.95	0.82	−1.53	2.27×10^{-33}	8.91×10^{-31}
PAM16	51025	16p13.3	8.07	6.78	0.81	0.68	1.29	3.64×10^{-33}	1.39×10^{-30}
SPTLC2	9517	14q24.3	9.76	10.76	0.63	0.55	−1	9.88×10^{-33}	3.20×10^{-30}
HMBOX1	79618	8p21.1–p12	5.77	6.85	0.69	0.71	−1.09	2.28×10^{-32}	6.21×10^{-30}
DDX21	9188	10q22.1	9.2	10.55	0.86	0.55	−1.35	2.79×10^{-32}	7.08×10^{-30}
ELK4	2005	1q32.1	6.52	7.59	0.68	0.61	−1.07	3.38×10^{-32}	8.10×10^{-30}
MAN1A2	10905	1p12	8.4	9.68	0.82	0.59	−1.28	3.26×10^{-32}	8.10×10^{-30}
MINDY2	54629	15q21.3–q22.1	7.06	8.49	0.91	0.78	−1.43	3.55×10^{-32}	8.35×10^{-30}
DPP8	54878	15q22.31	7.61	8.91	0.84	0.52	−1.31	4.59×10^{-32}	1.04×10^{-29}
PANK3	79646	5q34	7.63	8.76	0.72	0.58	−1.13	4.71×10^{-32}	1.05×10^{-29}
ETV3	2117	1q23.1	5.4	7.26	1.19	0.72	−1.86	5.48×10^{-32}	1.16×10^{-29}
LRRC29	26231	16q22.1	7.54	6.47	0.7	0.64	1.08	1.06×10^{-31}	2.18×10^{-29}
TP53I13	90313	17q11.2	10.04	9.06	0.63	0.66	0.98	1.86×10^{-31}	3.59×10^{-29}
MYCBP2	23077	13q22.3	9.52	10.55	0.68	0.54	−1.04	2.96×10^{-31}	5.32×10^{-29}
UHMK1	127933	1q23.3	6.74	8.93	1.44	0.98	−2.19	3.60×10^{-31}	6.37×10^{-29}
SKIL	6498	3q26.2	8.04	9.2	0.76	0.67	−1.16	6.40×10^{-31}	1.06×10^{-28}
THAP3	90326	1p36.31	8.93	7.91	0.68	0.59	1.02	8.84×10^{-31}	1.44×10^{-28}
TOR1AIP2	163590	1q25.2	7.66	8.86	0.8	0.54	−1.2	9.19×10^{-31}	1.47×10^{-28}
KMT2E−AS1	100216545	7q22.3	7.61	6.31	0.85	0.91	1.3	1.15×10^{-30}	1.79×10^{-28}
CERS6	253782	2q24.3	8.38	9.53	0.76	0.66	−1.15	1.50×10^{-30}	2.24×10^{-28}
ASXL2	55252	2p23.3	7.74	9.54	1.2	0.72	−1.8	1.70×10^{-30}	2.48×10^{-28}
TRIP11	9321	14q32.12	8.48	9.5	0.69	0.52	−1.03	2.09×10^{-30}	2.93×10^{-28}
GPX4	2879	19p13.3	12.74	11.66	0.72	0.66	1.08	2.22×10^{-30}	3.09×10^{-28}
PFDN6	10471	6p21.32	9.56	8.57	0.66	0.52	0.99	2.25×10^{-30}	3.09×10^{-28}
POLR2J	5439	7q22.1	10.54	9.54	0.67	0.58	1	2.35×10^{-30}	3.17×10^{-28}
TRIM44	54765	11p13	9.37	10.64	0.85	0.55	−1.27	2.34×10^{-30}	3.17×10^{-28}
FAM168A	23201	11q13.4	6.56	8.08	1.01	0.65	−1.51	2.62×10^{-30}	3.49×10^{-28}
PPP1R16A	84988	8q24.3	10.57	9.56	0.66	0.81	1.01	2.87×10^{-30}	3.75×10^{-28}
AURKAIP1	54998	1p36.33	10.88	9.85	0.7	0.63	1.04	4.08×10^{-30}	5.19×10^{-28}
NDUFA7	4701	19p13.2	10.12	9.08	0.7	0.57	1.04	5.01×10^{-30}	6.17×10^{-28}
PIK3CG	5294	7q22.3	5.95	7.63	1.14	1.05	−1.68	6.60×10^{-30}	7.91×10^{-28}
SLC30A1	7779	1q32.3	7.41	8.73	0.89	0.7	−1.31	7.33×10^{-30}	8.63×10^{-28}
WWTR1	25937	3q25.1	9.39	10.59	0.82	0.63	−1.2	7.84×10^{-30}	9.14×10^{-28}
DGKH	160851	13q14.11	4.72	6.34	1.1	0.98	−1.62	9.20×10^{-30}	1.05×10^{-27}
FMC1	154791	7q34	8.68	7.68	0.68	0.63	1.01	1.01×10^{-29}	1.13×10^{-27}
ADAM10	102	15q21.3	9.98	11.31	0.91	0.48	−1.33	1.01×10^{-29}	1.13×10^{-27}
UQCC2	84300	6p21.31	9.83	8.76	0.73	0.63	1.07	1.15×10^{-29}	1.27×10^{-27}
FDX2	112812	19p13.2	8.27	7.27	0.69	0.59	1.01	1.19×10^{-29}	1.30×10^{-27}
THAP7	80764	22q11.21	8.97	7.98	0.68	0.61	0.99	1.47×10^{-29}	1.55×10^{-27}
MAP3K2	10746	2q14.3	8.22	9.45	0.84	0.53	−1.23	1.79×10^{-29}	1.81×10^{-27}
LMBRD2	92255	5p13.2	6.29	7.86	1.08	0.81	−1.56	2.26×10^{-29}	2.23×10^{-27}
NDUFS7	374291	19p13.3	10.52	9.44	0.74	0.71	1.09	2.30×10^{-29}	2.24×10^{-27}
ITGA9	3680	3p22.2	6.14	8.1	1.35	1.06	−1.97	2.31×10^{-29}	2.24×10^{-27}
NDUFS8	4728	11q13.2	10.81	9.55	0.87	0.76	1.26	2.84×10^{-29}	2.66×10^{-27}
WASH2P	375260	2q14.1	8.99	8.04	0.65	0.68	0.96	2.96×10^{-29}	2.76×10^{-27}
ELK3	2004	12q23.1	8.03	9.61	1.09	0.72	−1.58	3.03×10^{-29}	2.80×10^{-27}
KIF13A	63971	6p22.3	8.64	9.79	0.8	0.51	−1.15	4.69×10^{-29}	4.19×10^{-27}

MIEN1	84299	17q12	10.15	9.17	0.68	0.55	0.98	5.17×10^{-29}	4.55×10^{-27}
HIPK3	10114	11p13	8.79	10.55	1.22	0.62	−1.75	5.30×10^{-29}	4.61×10^{-27}
ROCK2	9475	2p25.1	8.7	10.12	0.99	0.54	−1.42	5.52×10^{-29}	4.76×10^{-27}
ERCC6L2	375748	9q22.32	5.39	6.96	1.09	0.68	−1.57	5.94×10^{-29}	5.06×10^{-27}
SSNA1	8636	9q34.3	10.11	9.14	0.67	0.65	0.96	6.65×10^{-29}	5.56×10^{-27}
ANKRD50	57182	4q28.1	8.67	9.68	0.7	0.59	−1	6.85×10^{-29}	5.68×10^{-27}
IL6ST	3572	5q11.2	8.84	11.11	1.59	0.88	−2.28	7.94×10^{-29}	6.55×10^{-27}
MCRIP2	84331	16p13.3	10.14	8.72	0.99	0.96	1.43	8.54×10^{-29}	7.00×10^{-27}
ZNHIT1	10467	7q22.1	11.14	10.05	0.77	0.58	1.09	9.45×10^{-29}	7.69×10^{-27}
DGCR6	8214	22q11.21|22q11	9.68	8.61	0.75	0.74	1.07	1.28×10^{-28}	1.00×10^{-26}
PAXX	286257	9q34.3	8.83	7.67	0.81	0.75	1.16	1.36×10^{-28}	1.06×10^{-26}
LIMS1	3987	2q12.3	7.6	9.13	1.08	0.68	−1.53	1.35×10^{-28}	1.06×10^{-26}
FER	2241	5q21.3	5.04	6.22	0.83	0.69	−1.18	1.42×10^{-28}	1.10×10^{-26}
NUDT8	254552	11q13.2	7.89	6.54	0.95	0.87	1.35	2.14×10^{-28}	1.59×10^{-26}
C19ORF53	28974	19p13.13	11.2	10.18	0.72	0.58	1.02	2.20×10^{-28}	1.61×10^{-26}
ALKBH6	84964	19q13.12	7.37	6.42	0.67	0.62	0.95	2.23×10^{-28}	1.62×10^{-26}
GADD45GIP1	90480	19p13.13	10.66	9.46	0.85	0.68	1.2	2.79×10^{-28}	1.99×10^{-26}
BROX	148362	1q41	7.05	8.46	1	0.59	−1.41	3.68×10^{-28}	2.56×10^{-26}
ELOB	6923	16p13.3	11.58	10.44	0.81	0.71	1.14	4.40×10^{-28}	2.98×10^{-26}
BLOC1S1	2647	12q13.2	11.12	10.03	0.78	0.53	1.09	5.07×10^{-28}	3.37×10^{-26}
MRPS15	64960	1p34.3	10.57	9.6	0.69	0.5	0.97	5.69×10^{-28}	3.67×10^{-26}
SCO2	9997	22q13.33	9.73	8.76	0.69	0.68	0.97	7.55×10^{-28}	4.68×10^{-26}
FKBP2	2286	11q13.1	10.96	9.92	0.75	0.65	1.04	9.49×10^{-28}	5.79×10^{-26}
BAD	572	11q13.1	10.55	9.59	0.68	0.57	0.95	1.03×10^{-27}	6.22×10^{-26}
CDK10	8558	16q24.3	10.96	9.98	0.69	0.76	0.97	1.05×10^{-27}	6.29×10^{-26}
YDJC	150223	22q11.21	9.16	8.05	0.8	0.68	1.11	1.07×10^{-27}	6.40×10^{-26}
SCAND1	51282	20q11.23	10.26	9.09	0.83	0.88	1.17	1.15×10^{-27}	6.80×10^{-26}
LPP	4026	3q27.3–q28	8.08	9.16	0.77	0.74	−1.08	1.17×10^{-27}	6.87×10^{-26}
MYO9A	4649	15q23	8.54	9.96	1.02	0.83	−1.42	1.17×10^{-27}	6.87×10^{-26}
NDC1	55706	1p32.3	6.65	7.71	0.77	0.54	−1.06	1.56×10^{-27}	8.73×10^{-26}
MXD3	83463	5q35.3	8.29	7	0.93	0.77	1.29	1.59×10^{-27}	8.76×10^{-26}
REST	5978	4q12	6.64	8.33	1.23	0.67	−1.69	1.73×10^{-27}	9.45×10^{-26}
ST20	400410	15q25.1	6.68	5.62	0.77	0.76	1.07	3.02×10^{-27}	1.55×10^{-25}
PAFAH1B2	5049	11q23.3	7.71	9.08	1.01	0.51	−1.37	3.17×10^{-27}	1.62×10^{-25}
P2RY1	5028	3q25.2	4.74	6.67	1.41	1.1	−1.93	3.26×10^{-27}	1.65×10^{-25}
MRPL55	128308	1q42.13	9.81	8.7	0.81	0.64	1.11	3.62×10^{-27}	1.81×10^{-25}
ROMO1	140823	20q11.22	10.35	9.08	0.92	0.75	1.26	3.67×10^{-27}	1.83×10^{-25}
SLX1B	79008	16p11.2	9.73	8.72	0.74	0.63	1.01	3.83×10^{-27}	1.89×10^{-25}
NDUFA3	4696	19q13.42	10.33	9.04	0.94	0.81	1.29	4.50×10^{-27}	2.20×10^{-25}
DICER1−AS1	400242	14q32.13	6.53	5.22	0.94	0.96	1.3	4.80×10^{-27}	2.32×10^{-25}
GPRIN3	285513	4q22.1	7.14	8.78	1.2	0.9	−1.64	4.83×10^{-27}	2.33×10^{-25}
FBXL6	26233	8q24.3	8.62	7.55	0.78	0.76	1.07	6.28×10^{-27}	2.97×10^{-25}
ECI1	1632	16p13.3	11.04	9.97	0.79	0.73	1.07	6.32×10^{-27}	2.97×10^{-25}
PSENEN	55851	19q13.12	10.47	9.52	0.7	0.56	0.95	7.00×10^{-27}	3.25×10^{-25}
MAN2A1	4124	5q21.3	8.34	9.97	1.21	0.76	−1.64	7.39×10^{-27}	3.38×10^{-25}
RNF168	165918	3q29	5.9	7.22	0.98	0.55	−1.32	7.67×10^{-27}	3.48×10^{-25}
NDUFB7	4713	19p13.12	11.21	10.04	0.86	0.79	1.18	8.02×10^{-27}	3.59×10^{-25}
LCOR	84458	10q24.1	5.21	6.66	1.07	0.83	−1.45	8.11×10^{-27}	3.62×10^{-25}
C10ORF12	84458	10q24.1	5.37	6.41	0.77	0.58	−1.04	8.15×10^{-27}	3.63×10^{-25}
PTPRG	5793	3p14.2	9.3	10.61	0.97	0.76	−1.31	9.56×10^{-27}	4.21×10^{-25}
SLC30A6	55676	2p22.3	7.3	8.36	0.79	0.52	−1.06	1.39×10^{-26}	5.93×10^{-25}
ARL5B	221079	10p12.31	6.87	8.01	0.85	0.68	−1.15	1.42×10^{-26}	6.03×10^{-25}
PARD3B	117583	2q33.3	4.42	6.37	1.45	0.93	−1.95	1.51×10^{-26}	6.37×10^{-25}
DDR2	4921	1q23.3	5.26	7.42	1.61	1.13	−2.16	1.59×10^{-26}	6.63×10^{-25}
TLR4	7099	9q33.1	8.98	9.99	0.75	0.67	−1.01	1.77×10^{-26}	7.31×10^{-25}
IPMK	253430	10q21.1	4.52	6.35	1.37	0.86	−1.83	1.79×10^{-26}	7.39×10^{-25}
TCAF1	9747	7q35	6.9	8.35	1.09	0.71	−1.45	2.27×10^{-26}	9.21×10^{-25}
TMEM256	254863	17p13.1	9.77	8.82	0.71	0.56	0.95	2.94×10^{-26}	1.15×10^{-24}
MRPL23	6150	11p15.5	10.44	9.36	0.81	0.62	1.08	3.22×10^{-26}	1.25×10^{-24}
WASH7P	653635	1p36.33	10.85	9.79	0.79	0.79	1.06	3.46×10^{-26}	1.33×10^{-24}
MPST	4357	22q12.3	10.68	9.62	0.8	0.68	1.07	3.60×10^{-26}	1.38×10^{-24}
QTRT1	81890	19p13.2	10.02	9.02	0.74	0.77	0.99	3.73×10^{-26}	1.43×10^{-24}
UTRN	7402	6q24.2	10.84	11.88	0.79	0.64	−1.04	3.81×10^{-26}	1.45×10^{-24}
WASH5P	375690	19p13.3	8.26	7.25	0.76	0.7	1.01	4.15×10^{-26}	1.56×10^{-24}
RIF1	55183	2q23.3	8.2	9.7	1.14	0.64	−1.5	4.32×10^{-26}	1.62×10^{-24}
SLC30A4	7782	15q21.1|15q21.1	5.51	6.98	1.11	0.65	−1.47	4.37×10^{-26}	1.63×10^{-24}
TGFBR2	7048	3p24.1	11.5	12.7	0.91	0.67	−1.21	4.62×10^{-26}	1.71×10^{-24}
EYA3	2140	1p35.3	5.52	7.04	1.15	0.73	−1.52	4.91×10^{-26}	1.80×10^{-24}
PDZD8	118987	10q25.3–q26.11	8.14	9.43	0.98	0.68	−1.29	5.58×10^{-26}	2.02×10^{-24}
NDUFA2	4695	5q31.3	11.13	10	0.86	0.62	1.13	6.18×10^{-26}	2.23×10^{-24}
FAAP20	199990	1p36.33	9.18	8.21	0.73	0.7	0.97	6.27×10^{-26}	2.25×10^{-24}

CAMK1D	57118	10p13	5.55	6.74	0.9	0.65	−1.19	6.91×10^{-26}	2.46×10^{-24}
REX1BD	55049	19p13.11	9.2	8.07	0.86	0.77	1.14	7.87×10^{-26}	2.76×10^{-24}
HSD17B14	51171	19q13.33	10.16	9.04	0.82	1.05	1.12	8.57×10^{-26}	2.98×10^{-24}
COX5B	1329	2q11.2	11.99	10.93	0.8	0.64	1.05	9.56×10^{-26}	3.32×10^{-24}
CREBRF	153222	5q35.1	8.54	9.61	0.82	0.63	−1.07	1.07×10^{-25}	3.67×10^{-24}
CDYL2	124359	16q23.2	5.23	6.68	1.1	1.01	−1.45	1.17×10^{-25}	4.00×10^{-24}
KCTD20	222658	6p21.31	9.64	10.75	0.86	0.42	−1.11	1.45×10^{-25}	4.89×10^{-24}
ZNF366	167465	5q13.2|5q13.2	4.81	6.91	1.62	1.21	−2.1	2.60×10^{-25}	8.22×10^{-24}
PRKAR2A	5576	3p21.31	6.19	7.93	1.35	0.67	−1.74	3.00×10^{-25}	9.45×10^{-24}
LSM7	51690	19p13.3	9.12	8.14	0.76	0.65	0.98	3.15×10^{-25}	9.91×10^{-24}
HEG1	57493	3q21.2	10.65	11.73	0.83	0.76	−1.09	3.16×10^{-25}	9.91×10^{-24}
PGM2L1	283209	11q13.4	8.21	9.23	0.79	0.7	−1.02	3.29×10^{-25}	1.03×10^{-23}
CARNMT1	138199	9q21.13	5.79	7.33	1.2	0.6	−1.54	3.53×10^{-25}	1.09×10^{-23}
CCDC84	338657	11q23.3	7.71	6.53	0.91	0.91	1.19	3.81×10^{-25}	1.18×10^{-23}
MISP3	113230	19p13.12	7.51	6.21	0.99	1.03	1.3	4.41×10^{-25}	1.35×10^{-23}
USP12	219333	13q12.13	7.33	8.83	1.17	0.58	−1.5	4.82×10^{-25}	1.46×10^{-23}
PYCR3	65263	8q24.3	8.72	7.76	0.74	0.72	0.96	6.37×10^{-25}	1.90×10^{-23}
MRPL41	64975	9q34.3	9.76	8.43	1.04	0.83	1.33	6.64×10^{-25}	1.97×10^{-23}
IDE	3416	10q23.33	7.93	8.91	0.77	0.51	−0.98	6.74×10^{-25}	2.00×10^{-23}
ANKRD36BP1	84832	1q24.2	5.6	7.4	1.42	0.84	−1.81	7.20×10^{-25}	2.12×10^{-23}
ZNF134	7693	19q13.43	7.01	7.97	0.75	0.52	−0.96	7.38×10^{-25}	2.16×10^{-23}
H2AFJ	55766	12p12.3	10.68	9.7	0.77	0.64	0.98	9.07×10^{-25}	2.60×10^{-23}
CCDC186	55088	10q25.3	7.6	8.75	0.91	0.61	−1.16	9.03×10^{-25}	2.60×10^{-23}
TIAF1	9220	17q11.2	9.21	8.2	0.79	0.63	1.01	9.85×10^{-25}	2.80×10^{-23}
PRKG1	5592	10q11.23–q21.1	5.22	7.2	1.56	1.17	−1.98	1.13×10^{-24}	3.15×10^{-23}
MIGA1	374986	1p31.1	8.63	9.69	0.83	0.61	−1.05	1.25×10^{-24}	3.48×10^{-23}
ATE1	11101	10q26.13	6.51	8.05	1.22	0.6	−1.54	1.28×10^{-24}	3.56×10^{-23}
EXOC6B	23233	2p13.2	5.86	7.47	1.28	0.65	−1.61	1.29×10^{-24}	3.57×10^{-23}
KLHDC10	23008	7q32.2	9.1	10.25	0.91	0.67	−1.15	1.31×10^{-24}	3.61×10^{-23}
TTBK2	146057	15q15.2	6.07	7.35	1.01	0.71	−1.28	1.33×10^{-24}	3.67×10^{-23}
DCXR	51181	17q25.3	9.7	8.44	0.99	0.8	1.26	1.41×10^{-24}	3.84×10^{-23}
ZNF837	116412	19q13.43	5.83	4.85	0.75	0.85	0.97	1.43×10^{-24}	3.88×10^{-23}
COX6B1	1340	19q13.12	12.19	11.22	0.76	0.56	0.96	1.71×10^{-24}	4.59×10^{-23}
ANKS3	124401	16p13.3	9.5	8.52	0.77	0.68	0.98	2.05×10^{-24}	5.38×10^{-23}
N4BP2	55728	4p14	5.5	7.08	1.26	0.77	−1.58	2.06×10^{-24}	5.39×10^{-23}
DAAM1	23002	14q23.1	6.48	7.74	1	0.7	−1.26	2.35×10^{-24}	6.04×10^{-23}
APOOL	139322	Xq21.1	6.22	7.7	1.18	0.75	−1.48	2.39×10^{-24}	6.10×10^{-23}
TMEM191A	84222	22q11.21	4.96	3.82	0.9	0.82	1.14	2.46×10^{-24}	6.26×10^{-23}
ENKD1	84080	16q22.1	9.46	8.45	0.8	0.67	1.01	2.65×10^{-24}	6.73×10^{-23}
ABHD16B	140701	20q13.33	7.62	6.63	0.78	0.78	1	3.01×10^{-24}	7.53×10^{-23}
ATP5ME	521	4p16.3	11.26	10.19	0.85	0.63	1.07	3.04×10^{-24}	7.61×10^{-23}
BAG4	9530	8p11.23	6.43	7.46	0.83	0.49	−1.03	3.21×10^{-24}	7.98×10^{-23}
ETS1	2113	11q24.3	11.7	12.79	0.87	0.74	−1.09	3.76×10^{-24}	9.24×10^{-23}
LYSMD4	145748	15q26.3	8.34	7.36	0.79	0.57	0.98	3.81×10^{-24}	9.31×10^{-23}
RNF169	254225	11q13.4	8.23	9.29	0.85	0.46	−1.06	4.06×10^{-24}	9.85×10^{-23}
SERF2	10169	15q15.3	13.56	12.55	0.81	0.57	1.01	4.13×10^{-24}	9.99×10^{-23}
EIF4EBP3	8637	5q31.3	10.11	9.12	0.79	0.68	0.99	4.27×10^{-24}	1.03×10^{-22}
SLC25A24	29957	1p13.3	9	10.03	0.83	0.44	−1.03	4.48×10^{-24}	1.08×10^{-22}
MCTP1	79772	5q15	5.53	7.11	1.27	0.93	−1.58	5.60×10^{-24}	1.32×10^{-22}
MICOS13	125988	19p13.3	10.58	9.53	0.85	0.61	1.05	6.11×10^{-24}	1.42×10^{-22}
EGFR	1956	7p11.2	10.14	11.47	1.06	1.06	−1.33	7.48×10^{-24}	1.71×10^{-22}
LIN7B	64130	19q13.33	5.51	4.31	0.95	0.96	1.2	7.73×10^{-24}	1.76×10^{-22}
NAA38	84316	17p13.1	9.68	8.68	0.81	0.66	1	8.75×10^{-24}	1.96×10^{-22}
GTF2H3	2967	12q24.31	6.46	7.54	0.88	0.49	−1.08	9.31×10^{-24}	2.08×10^{-22}
CSNK2A3	283106	11p15.4	7.68	8.87	0.98	0.52	−1.19	1.29×10^{-23}	2.78×10^{-22}
TAF10	6881	11p15.4	10.2	9.17	0.84	0.68	1.04	1.30×10^{-23}	2.79×10^{-22}
SAC3D1	29901	11q13.1	7.24	6.29	0.77	0.7	0.95	1.54×10^{-23}	3.29×10^{-22}
ZBTB10	65986	8q21.13	8.23	9.29	0.86	0.7	−1.06	1.65×10^{-23}	3.48×10^{-22}
RASSF7	8045	11p15.5	10.39	9.27	0.9	0.85	1.12	1.67×10^{-23}	3.50×10^{-22}
RAD54L2	23132	3p21.2	5.72	7.16	1.18	0.76	−1.44	2.02×10^{-23}	4.21×10^{-22}
RPS6KA5	9252	14q32.11	5.72	6.84	0.92	0.66	−1.12	2.11×10^{-23}	4.38×10^{-22}
NCOA2	10499	8q13.3	7.66	9.44	1.47	0.8	−1.78	2.35×10^{-23}	4.85×10^{-22}
PDE3A	5139	12p12.2	5.91	8.06	1.77	1.14	−2.14	2.77×10^{-23}	5.59×10^{-22}
NDUFA13	51079	19p13.11	12.11	11.06	0.87	0.6	1.05	2.80×10^{-23}	5.64×10^{-22}
MIF	4282	22q11.23	13.85	12.61	0.99	1.1	1.24	3.15×10^{-23}	6.28×10^{-22}
PPM1L	151742	3q25.33–q26.1	5.64	7.11	1.2	1.03	−1.47	3.38×10^{-23}	6.70×10^{-22}
BRAF	673	7q34	6.44	7.5	0.88	0.58	−1.06	3.43×10^{-23}	6.79×10^{-22}
CYSLTR1	10800	Xq21.1	4.61	6.03	1.18	0.85	−1.42	3.75×10^{-23}	7.41×10^{-22}
COX6C	1345	8q22.2	11.42	10.45	0.81	0.61	0.98	3.94×10^{-23}	7.74×10^{-22}
ITGA1	3672	5q11.2	10.59	11.77	0.97	0.79	−1.18	4.05×10^{-23}	7.95×10^{-22}
CEMP1	752014	16p13.3	8.83	7.78	0.86	0.75	1.05	4.18×10^{-23}	8.19×10^{-22}

WASH8P	100288778	12p13.33	9.47	8.38	0.89	0.79	1.09	5.42 x 10^{-23}	1.05 x 10^{-21}
KMT5C	84787	19q13.42	7.24	6.23	0.83	0.75	1.01	6.41 x 10^{-23}	1.22 x 10^{-21}
ROR1	4919	1p31.3	4.73	6.32	1.33	0.95	−1.59	6.40 x 10^{-23}	1.22 x 10^{-21}
ZNF593	51042	1p36.11	8.46	7.41	0.88	0.75	1.06	8.51 x 10^{-23}	1.60 x 10^{-21}
MFHAS1	9258	8p23.1	6.77	8.28	1.27	0.84	−1.5	1.11 x 10^{-22}	2.04 x 10^{-21}
ZNF689	115509	16p11.2	6.22	7.23	0.85	0.44	−1.01	1.13 x 10^{-22}	2.09 x 10^{-21}
PPP4R2	151987	3p13	7.19	8.33	0.96	0.49	−1.14	1.14 x 10^{-22}	2.10 x 10^{-21}
KLHL11	55175	17q21.2	3.97	5.51	1.3	0.76	−1.54	1.17 x 10^{-22}	2.15 x 10^{-21}
8–Mar	220972	10q11.21–q11.22	7.19	8.47	1.09	0.62	−1.28	1.29 x 10^{-22}	2.36 x 10^{-21}
PPDPF	79144	20q13.33	12.45	11.43	0.85	0.8	1.02	1.34 x 10^{-22}	2.43 x 10^{-21}
ITGA2	3673	5q11.2	8.66	9.93	1.07	0.76	−1.26	1.34 x 10^{-22}	2.43 x 10^{-21}
KIFC2	90990	8q24.3	9.18	7.83	1.13	1.03	1.35	1.58 x 10^{-22}	2.84 x 10^{-21}
MOSPD2	158747	Xp22.2	7.29	8.3	0.86	0.47	−1.01	1.59 x 10^{-22}	2.85 x 10^{-21}
TMPPE	643853	3p22.3	4.07	5.2	0.95	0.73	−1.13	1.83 x 10^{-22}	3.24 x 10^{-21}
COX4I1	1327	16q24.1	13.17	12.18	0.83	0.59	0.99	1.87 x 10^{-22}	3.30 x 10^{-21}
TMTC1	83857	12p11.22	9.18	10.4	1.02	0.9	−1.21	2.25 x 10^{-22}	3.95 x 10^{-21}
METTL26	84326	16p13.3	10.93	9.78	0.97	0.79	1.15	3.29 x 10^{-22}	5.60 x 10^{-21}
HIP1	3092	7q11.23	9.44	10.59	0.99	0.6	−1.15	3.30 x 10^{-22}	5.63 x 10^{-21}
RSPRY1	89970	16q13	7.93	8.95	0.88	0.43	−1.02	3.36 x 10^{-22}	5.71 x 10^{-21}
SAPCD1	401251	6p21.33	6.44	5.17	1.07	1.01	1.28	3.68 x 10^{-22}	6.20 x 10^{-21}
ZNF426	79088	19p13.2	5.52	6.8	1.09	0.8	−1.28	3.67 x 10^{-22}	6.20 x 10^{-21}
UGCG	7357	9q31.3	7.92	9.19	1.09	0.69	−1.26	4.82 x 10^{-22}	7.95 x 10^{-21}
ZBTB37	84614	1q25.1	3.62	4.77	0.99	0.73	−1.15	5.18 x 10^{-22}	8.51 x 10^{-21}
ZNF430	80264	19p12	5.78	6.76	0.84	0.47	−0.97	5.66 x 10^{-22}	9.24 x 10^{-21}
NECAB3	63941	20q11.22	8.8	7.82	0.83	0.78	0.98	5.90 x 10^{-22}	9.57 x 10^{-21}
NDUFAF8	284184	17q25.3	8.65	7.67	0.84	0.64	0.98	6.66 x 10^{-22}	1.07 x 10^{-20}
ITGA4	3676	2q31.3	8.66	9.95	1.11	0.8	−1.29	7.62 x 10^{-22}	1.21 x 10^{-20}
PUSL1	126789	1p36.33	7.44	6.41	0.88	0.82	1.03	7.92 x 10^{-22}	1.26 x 10^{-20}
NSUN5P1	155400	7q11.23	7.4	5.85	1.27	1.56	1.54	8.06 x 10^{-22}	1.28 x 10^{-20}
KBTBD7	84078	13q14.11	5.92	7.29	1.19	0.71	−1.37	9.95 x 10^{-22}	1.55 x 10^{-20}
NUPR1	26471	16p11.2	12.38	10.93	1.25	0.98	1.45	1.12 x 10^{-21}	1.73 x 10^{-20}
CCDC107	203260	9p13.3	9.89	8.88	0.87	0.69	1	1.46 x 10^{-21}	2.22 x 10^{-20}
FRMD3	257019	9q21.32	6.29	8.18	1.64	1.32	−1.89	1.77 x 10^{-21}	2.66 x 10^{-20}
COX6A1	1337	12q24.31|12q24.2	11.93	10.9	0.9	0.64	1.03	1.84 x 10^{-21}	2.74 x 10^{-20}
HSD11B1L	374875	19p13.3	7.47	6.51	0.84	0.71	0.96	1.91 x 10^{-21}	2.84 x 10^{-20}
SLC8A1	6546	2p22.1	8.35	9.46	0.96	0.88	−1.11	2.05 x 10^{-21}	3.03 x 10^{-20}
KLF7	8609	2q33.3	6.36	7.67	1.15	0.75	−1.31	2.08 x 10^{-21}	3.08 x 10^{-20}
LEPROT	54741	1p31.3	8.68	10.2	1.35	0.61	−1.52	2.24 x 10^{-21}	3.30 x 10^{-20}
PRR5	55615	22q13.31	8.59	7.63	0.83	0.73	0.96	2.31 x 10^{-21}	3.38 x 10^{-20}
SESN3	143686	11q21	5.33	6.83	1.31	1.03	−1.5	2.45 x 10^{-21}	3.58 x 10^{-20}
LUC7L	55692	16p13.3	9.84	8.82	0.88	0.88	1.02	2.74 x 10^{-21}	3.98 x 10^{-20}
ANKRD44	91526	2q33.1	5.9	7.2	1.14	0.85	−1.29	2.76 x 10^{-21}	4.01 x 10^{-20}
SLK	9748	10q24.33–q25.1	9.89	10.85	0.85	0.48	−0.96	2.93 x 10^{-21}	4.23 x 10^{-20}
ATF2	1386	2q31.1	8.06	9.23	1.04	0.46	−1.17	3.03 x 10^{-21}	4.36 x 10^{-20}
CPED1	79974	7q31.31	7.16	8.52	1.19	0.96	−1.36	3.23 x 10^{-21}	4.63 x 10^{-20}
SLC25A10	1468	17q25.3	9.73	8.69	0.9	0.87	1.04	3.55 x 10^{-21}	5.06 x 10^{-20}
NSUN5P2	260294	7q11.23	8.75	7.31	1.21	1.49	1.45	3.75 x 10^{-21}	5.32 x 10^{-20}
CDK2AP2	10263	11q13.2	11.43	10.47	0.84	0.78	0.97	3.97 x 10^{-21}	5.61 x 10^{-20}
STT3B	201595	3p23	9.11	10.25	1.02	0.49	−1.14	4.09 x 10^{-21}	5.75 x 10^{-20}
HSPB1	3315	7q11.23	13.92	12.8	0.97	0.93	1.11	4.17 x 10^{-21}	5.86 x 10^{-20}
ERN1	2081	17q23.3	5.96	7.49	1.36	0.83	−1.52	5.14 x 10^{-21}	7.13 x 10^{-20}
PRR22	163154	19p13.3	6.15	4.87	1.12	1.08	1.28	5.61 x 10^{-21}	7.70 x 10^{-20}
MAML2	23373	11q21	8.38	9.83	1.3	0.74	−1.45	5.70 x 10^{-21}	7.81 x 10^{-20}
RALGAPA2	57186	20p11.23	7.19	8.71	1.35	0.93	−1.52	5.87 x 10^{-21}	8.01 x 10^{-20}
CD93	22918	20p11.21	11.4	12.6	1.06	0.92	−1.2	5.89 x 10^{-21}	8.03 x 10^{-20}
ALKBH7	84266	19p13.3	9.96	8.98	0.87	0.73	0.98	6.64 x 10^{-21}	8.99 x 10^{-20}
CAPS	828	19p13.3	7.63	6.18	1.27	1.2	1.45	6.98 x 10^{-21}	9.37 x 10^{-20}
ATP5MF	9551	7q22.1	11.11	10.12	0.88	0.71	0.99	7.72 x 10^{-21}	1.03 x 10^{-19}
PRADC1	84279	2p13.2	8.74	7.76	0.87	0.58	0.97	8.53 x 10^{-21}	1.13 x 10^{-19}
GASK1B	51313	4q32.1	9.94	11.16	1.07	0.97	−1.21	8.76 x 10^{-21}	1.16 x 10^{-19}
D2HGDH	728294	2q37.3	9.65	8.63	0.88	1.01	1.02	9.41 x 10^{-21}	1.24 x 10^{-19}
1–Mar	55016	4q32.2–q32.3	5.79	6.96	0.99	1.2	−1.16	9.54 x 10^{-21}	1.25 x 10^{-19}
SULT1A3	6818	16p11.2	9.71	8.71	0.88	0.87	1	1.00 x 10^{-20}	1.31 x 10^{-19}
IL7R	3575	5p13.2	5.1	7.16	1.85	1.38	−2.06	1.02 x 10^{-20}	1.34 x 10^{-19}
TM9SF2	9375	13q32.3	10.91	11.88	0.88	0.42	−0.97	1.06 x 10^{-20}	1.38 x 10^{-19}
PCSK4	54760	19p13.3	6.62	5.49	0.99	1.05	1.14	1.41 x 10^{-20}	1.81 x 10^{-19}
LOC728743	728743	7q36.1	8	6.93	0.96	0.78	1.07	1.53 x 10^{-20}	1.96 x 10^{-19}
KIAA1958	158405	9q32	5.73	6.83	0.99	0.88	−1.11	1.72 x 10^{-20}	2.18 x 10^{-19}
MSRB1	51734	16p13.3	10.8	9.84	0.85	0.83	0.96	2.04 x 10^{-20}	2.56 x 10^{-19}
FBN1	2200	15q21.1	9.85	11.04	1.07	0.98	−1.2	2.17 x 10^{-20}	2.70 x 10^{-19}

HPSE	10855	4q21.23	4.81	6.24	1.29	1.06	−1.43	2.28 x 10^{-20}	2.83 x 10^{-19}
CYBB	1536	Xp21.1–p11.4	9.33	10.68	1.2	1.25	−1.35	3.45 x 10^{-20}	4.18 x 10^{-19}
SLC22A18	5002	11p15.4	10.42	9.42	0.87	1	1	3.71 x 10^{-20}	4.47 x 10^{-19}
TMOD2	29767	15q21.2	8.52	9.49	0.88	0.79	−0.97	3.84 x 10^{-20}	4.62 x 10^{-19}
UEVLD	55293	11p15.1	7	8	0.92	0.46	−1	4.12 x 10^{-20}	4.92 x 10^{-19}
PTAFR	5724	1p35.3	5.69	7.15	1.31	1.24	−1.46	4.28 x 10^{-20}	5.10 x 10^{-19}
DDX60	55601	4q32.3	9.12	10.12	0.92	0.6	−1	4.79 x 10^{-20}	5.66 x 10^{-19}
CRACR2B	283229	11p15.5	8.66	7.41	1.11	1.17	1.25	5.15 x 10^{-20}	6.06 x 10^{-19}
RGP1	9827	9p13.3	6.38	7.54	1.07	0.7	−1.16	5.88 x 10^{-20}	6.83 x 10^{-19}
AKT3	10000	1q43–q44	9.49	10.45	0.88	0.65	−0.95	5.94 x 10^{-20}	6.89 x 10^{-19}
DOCK9	23348	13q32.3	9.98	10.97	0.9	0.8	−0.99	6.02 x 10^{-20}	6.98 x 10^{-19}
ANOS1	3730	Xp22.31	6.32	7.65	1.23	0.92	−1.33	6.54 x 10^{-20}	7.55 x 10^{-19}
KLHL17	339451	1p36.33	7.2	6.09	1	1.02	1.11	7.90 x 10^{-20}	9.02 x 10^{-19}
NIPAL2	79815	8q22.2	6.44	7.59	1.07	0.7	−1.15	9.20 x 10^{-20}	1.04 x 10^{-18}
HECW2	57520	2q32.3	8.06	9.36	1.18	1.15	−1.3	9.31 x 10^{-20}	1.05 x 10^{-18}
STARD10	10809	11q13.4	10.01	9.05	0.9	0.65	0.97	9.46 x 10^{-20}	1.07 x 10^{-18}
GUCY1A2	2977	11q22.3	5	6.32	1.22	1.06	−1.32	1.09 x 10^{-19}	1.23 x 10^{-18}
CCNI	10983	4q21.1	11.52	12.65	1.07	0.48	−1.13	1.20 x 10^{-19}	1.34 x 10^{-18}
ZFPM1	161882	16q24.2	6.66	5.64	0.93	0.93	1.02	1.25 x 10^{-19}	1.39 x 10^{-18}
MTLN	205251	2q13	8.53	7.36	1.09	0.82	1.17	1.28 x 10^{-19}	1.42 x 10^{-18}
PJVK	494513	2q31.2	5.16	4.05	1.02	0.88	1.1	1.41 x 10^{-19}	1.57 x 10^{-18}
UQCRQ	27089	5q31.1	11.7	10.62	1.02	0.69	1.08	1.76 x 10^{-19}	1.92 x 10^{-18}
XYLT1	64131	16p12.3	6.86	7.9	0.96	0.87	−1.04	2.09 x 10^{-19}	2.27 x 10^{-18}
PDE4B	5142	1p31.3	8.16	9.18	0.95	0.83	−1.02	2.16 x 10^{-19}	2.33 x 10^{-18}
TCF4	6925	18q21.2	10.2	11.28	1	0.89	−1.08	2.49 x 10^{-19}	2.67 x 10^{-18}
ENDOG	2021	9q34.11	8.17	7.11	0.99	0.88	1.06	2.51 x 10^{-19}	2.68 x 10^{-18}
UBXN11	91544	1p36.11	9.9	8.81	1.01	0.91	1.09	3.43 x 10^{-19}	3.60 x 10^{-18}
LOC100128288	100128288	17p13.1	6.34	5.27	0.99	1.02	1.08	3.54 x 10^{-19}	3.72 x 10^{-18}
PCDH17	27253	13q21.1	8.3	9.83	1.45	1.17	−1.54	3.72 x 10^{-19}	3.89 x 10^{-18}
GOLIM4	27333	3q26.2	9.25	10.49	1.19	0.48	−1.23	3.81 x 10^{-19}	3.98 x 10^{-18}
DIAPH2	1730	Xq21.33	7.78	8.75	0.92	0.66	−0.97	3.95 x 10^{-19}	4.11 x 10^{-18}
FUT11	170384	10q22.2	8.23	9.22	0.94	0.65	−0.98	4.84 x 10^{-19}	4.96 x 10^{-18}
MACROD1	28992	11q13.1	8.08	6.98	1.03	0.93	1.1	5.00 x 10^{-19}	5.10 x 10^{-18}
CD163	9332	12p13.31	9.21	10.65	1.33	1.4	−1.44	5.62 x 10^{-19}	5.69 x 10^{-18}
MZT2B	80097	2q21.1	11.15	10.18	0.92	0.85	0.98	5.75 x 10^{-19}	5.80 x 10^{-18}
PPP1R16B	26051	20q11.23	8.3	9.37	0.98	1.07	−1.07	5.83 x 10^{-19}	5.88 x 10^{-18}
SOCS7	30837	17q12	4.66	5.99	1.27	0.92	−1.33	6.07 x 10^{-19}	6.10 x 10^{-18}
ZNF692	55657	1q44	9.23	8.02	1.14	1	1.21	6.19 x 10^{-19}	6.21 x 10^{-18}
ATAD3B	83858	1p36.33	8.02	6.87	1.05	1.15	1.14	6.43 x 10^{-19}	6.43 x 10^{-18}
ARHGAP42	143872	11q22.1	8.45	9.62	1.13	0.72	−1.17	6.66 x 10^{-19}	6.66 x 10^{-18}
CHKB−CPT1B	386593	22q13.33	9.34	7.87	1.36	1.5	1.47	7.07 x 10^{-19}	7.04 x 10^{-18}
APBB3	10307	5q31.3	9.14	8.12	0.95	0.97	1.02	7.16 x 10^{-19}	7.13 x 10^{-18}
NPIPB3	23117	16p12.2	10.53	8.85	1.59	1.33	1.68	7.29 x 10^{-19}	7.24 x 10^{-18}
TST	7263	22q12.3	10.17	9.12	0.99	0.91	1.05	7.35 x 10^{-19}	7.29 x 10^{-18}
MRC1	4360	10p12.33	7.25	8.68	1.34	1.28	−1.43	7.79 x 10^{-19}	7.68 x 10^{-18}
CNTD1	124817	17q21.2–q21.31	5.25	4.19	0.99	1	1.05	9.61 x 10^{-19}	9.34 x 10^{-18}
FAM49A	81553	2p24.2	5.98	7.04	1.02	0.79	−1.06	1.01 x 10^{-18}	9.76 x 10^{-18}
PRR34−AS1	150381	22q13.31	6.88	5.79	1.04	0.89	1.09	1.08 x 10^{-18}	1.04 x 10^{-17}
KPNA5	3841	6q22.1	5.18	6.3	1.09	0.73	−1.12	1.27 x 10^{-18}	1.21 x 10^{-17}
JCAD	57608	10p11.23	10.14	11.14	0.95	0.93	−1	1.55 x 10^{-18}	1.47 x 10^{-17}
CCDC189	90835	16p11.2	6.06	4.92	1.08	1.08	1.14	1.66 x 10^{-18}	1.57 x 10^{-17}
LOC653653	653653	17q23.1	5.54	6.84	1.28	0.58	−1.3	1.67 x 10^{-18}	1.58 x 10^{-17}
FILIP1	27145	6q14.1	6.86	8.04	1.13	1.1	−1.19	1.68 x 10^{-18}	1.59 x 10^{-17}
CDK3	1018	17q25.1	8.08	6.93	1.1	1.05	1.15	1.81 x 10^{-18}	1.70 x 10^{-17}
ARNTL2	56938	12p11.23	3.87	5.36	1.44	1.15	−1.49	1.89 x 10^{-18}	1.78 x 10^{-17}
TNFRSF10A	8797	8p21.3	6.08	7.08	0.97	0.81	−1	2.06 x 10^{-18}	1.93 x 10^{-17}
PCYT2	5833	17q25.3	9.42	8.47	0.92	0.71	0.95	2.26 x 10^{-18}	2.11 x 10^{-17}
FAR2	55711	12p11.22	6.26	7.24	0.96	0.73	−0.98	2.51 x 10^{-18}	2.32 x 10^{-17}
FPR3	2359	19q13.41	8.25	9.57	1.28	1.1	−1.32	2.60 x 10^{-18}	2.40 x 10^{-17}
RCAN3	11123	1p36.11	5.06	6.23	1.16	0.84	−1.18	3.23 x 10^{-18}	2.94 x 10^{-17}
DNLZ	728489	9q34.3	7.28	6.19	1.07	0.86	1.09	3.61 x 10^{-18}	3.27 x 10^{-17}
CD226	10666	18q22.2	4.19	5.34	1.11	1.05	−1.15	3.70 x 10^{-18}	3.34 x 10^{-17}
ZNF813	126017	19q13.42	4.67	6.03	1.36	0.83	−1.36	3.97 x 10^{-18}	3.55 x 10^{-17}
HCFC1R1	54985	16p13.3	11.83	10.78	1.03	0.77	1.04	4.01 x 10^{-18}	3.60 x 10^{-17}
SCNN1D	6339	1p36.33	6.59	4.86	1.66	1.68	1.73	4.07 x 10^{-18}	3.64 x 10^{-17}
TMTC2	160335	12q21.31	8.02	9.14	1.1	0.82	−1.12	4.90 x 10^{-18}	4.34 x 10^{-17}
LRRC46	90506	17q21.32	4.86	3.7	1.14	1.06	1.17	5.06 x 10^{-18}	4.46 x 10^{-17}
TEK	7010	9p21.2	8.25	9.61	1.33	1.12	−1.36	5.06 x 10^{-18}	4.46 x 10^{-17}
CCDC28B	79140	1p35.2	6.97	5.89	1.06	0.95	1.08	5.32 x 10^{-18}	4.67 x 10^{-17}
GPR176	11245	15q14–q15.1	6.78	8.18	1.4	0.89	−1.4	6.22 x 10^{-18}	5.41 x 10^{-17}

GMCL1	64395	2p13.3	7.75	8.76	1.02	0.58	−1.01	6.51 x 10^{-18}	5.66 x 10^{-17}
GIMAP6	474344	7q36.1	9.67	10.65	0.96	0.8	−0.98	6.59 x 10^{-18}	5.72 x 10^{-17}
DPY19L3	147991	19q13.11	7.27	8.33	1.08	0.48	−1.06	7.19 x 10^{-18}	6.19 x 10^{-17}
FAM114A1	92689	4p14	8.04	8.99	0.96	0.54	−0.95	7.95 x 10^{-18}	6.81 x 10^{-17}
HMCN1	83872	1q25.3–q31.1	8.02	9.45	1.43	1.09	−1.43	1.31 x 10^{-17}	1.09 x 10^{-16}
SHE	126669	1q21.3	7.94	9.02	1.07	0.93	−1.08	1.42 x 10^{-17}	1.17 x 10^{-16}
LMTK2	22853	7q21.3	7.76	9	1.25	0.77	−1.23	1.41 x 10^{-17}	1.17 x 10^{-16}
C9ORF16	79095	9q34.11	9.41	8.42	1	0.81	1	2.17 x 10^{-17}	1.75 x 10^{-16}
FXYD2	486	11q23.3	14.01	12.53	1.44	1.62	1.48	2.41 x 10^{-17}	1.93 x 10^{-16}
TBC1D3B	414059	17q12	7.99	6.57	1.44	1.14	1.42	2.43 x 10^{-17}	1.94 x 10^{-16}
NT5M	56953	17p11.2	6.84	5.68	1.17	1.02	1.16	2.62 x 10^{-17}	2.08 x 10^{-16}
RECQL4	9401	8q24.3	6.88	5.87	1	1.02	1.01	2.67 x 10^{-17}	2.11 x 10^{-16}
ZNF436−AS1	148898	1p36.12	6.44	5.29	1.15	1.03	1.15	2.69 x 10^{-17}	2.13 x 10^{-16}
PLA2G6	8398	22q13.1	8.6	7.42	1.18	1.18	1.19	3.29 x 10^{-17}	2.58 x 10^{-16}
EMP1	2012	12p13.1	10.21	11.36	1.17	0.98	−1.15	3.31 x 10^{-17}	2.59 x 10^{-16}
PODXL	5420	7q32.3	11.64	12.7	1.07	0.99	−1.06	3.61 x 10^{-17}	2.81 x 10^{-16}
AGAP4	119016	10q11.22	8.23	7.05	1.19	1.05	1.18	3.66 x 10^{-17}	2.85 x 10^{-16}
OXER1	165140	2p21	7.61	6.39	1.24	1.03	1.22	4.09 x 10^{-17}	3.16 x 10^{-16}
HDAC9	9734	7p21.1	6.34	7.31	1	0.73	−0.97	4.34 x 10^{-17}	3.34 x 10^{-16}
FAM193B	54540	5q35.3	10.4	9.2	1.21	1.09	1.2	4.77 x 10^{-17}	3.64 x 10^{-16}
SPATA13	221178	13q12.12	7.9	9.08	1.22	0.8	−1.18	4.80 x 10^{-17}	3.67 x 10^{-16}
LINC01089	338799	12q24.31	8.45	7.26	1.17	1.34	1.19	4.84 x 10^{-17}	3.69 x 10^{-16}
CALHM5	254228	6q22.1	6.45	7.52	1.09	0.91	−1.07	5.45 x 10^{-17}	4.12 x 10^{-16}
BRCA2	675	13q13.1	5.04	6.24	1.26	0.85	−1.2	7.80 x 10^{-17}	5.78 x 10^{-16}
OSMR	9180	5p13.1	10.08	11.32	1.3	0.83	−1.24	8.19 x 10^{-17}	6.05 x 10^{-16}
SLC25A29	123096	14q32.2	9.12	8.16	0.98	0.9	0.96	8.27 x 10^{-17}	6.10 x 10^{-16}
SNHG10	283596	14q32.13	6.14	5.09	1.05	1.08	1.05	8.40 x 10^{-17}	6.19 x 10^{-16}
RBMS3	27303	3p24.1	5.32	6.55	1.28	0.93	−1.23	9.16 x 10^{-17}	6.73 x 10^{-16}
CYP7B1	9420	8q12.3	6.47	7.47	1.02	0.96	−1.01	9.50 x 10^{-17}	6.97 x 10^{-16}
HIST2H2AA3	8337	1q21.2	9.27	8.07	1.21	1.23	1.19	9.91 x 10^{-17}	7.25 x 10^{-16}
CCDC57	284001	17q25.3	9.01	8.03	0.98	1.02	0.97	1.04 x 10^{-16}	7.60 x 10^{-16}
TRIM56	81844	7q22.1	7.94	8.97	1.09	0.65	−1.03	1.06 x 10^{-16}	7.70 x 10^{-16}
SNX29	92017	16p13.13–p13.12	5.86	7.1	1.28	1.05	−1.23	1.24 x 10^{-16}	8.96 x 10^{-16}
NLN	57486	5q12.3	7.26	8.32	1.12	0.73	−1.06	1.38 x 10^{-16}	9.91 x 10^{-16}
STARD4	134429	5q22.1	6.65	7.61	1.02	0.66	−0.96	1.50 x 10^{-16}	1.07 x 10^{-15}
TLR7	51284	Xp22.2	6.79	8.01	1.22	1.32	−1.21	1.65 x 10^{-16}	1.17 x 10^{-15}
ZRANB3	84083	2q21.3	4.98	5.96	1.05	0.56	−0.99	1.72 x 10^{-16}	1.22 x 10^{-15}
SPACA6	147650	19q13.41	6.58	5.45	1.15	1.16	1.12	2.00 x 10^{-16}	1.41 x 10^{-15}
ARL15	54622	5q11.2	8.32	9.31	1.05	0.71	−0.99	2.14 x 10^{-16}	1.51 x 10^{-15}
SLX4IP	128710	20p12.2	3.56	4.62	1.13	0.8	−1.07	2.18 x 10^{-16}	1.53 x 10^{-15}
RAPGEF5	9771	7p15.3	9.63	10.65	1.08	0.85	−1.02	2.19 x 10^{-16}	1.54 x 10^{-15}
ZNF789	285989	7q22.1	7.22	6.25	1.03	0.79	0.97	2.28 x 10^{-16}	1.59 x 10^{-15}
PDCD1LG2	80380	9p24.1	5.59	6.61	1.05	1.04	−1.02	2.54 x 10^{-16}	1.77 x 10^{-15}
HCN3	57657	1q22	7.5	6.53	1.03	0.87	0.98	2.63 x 10^{-16}	1.83 x 10^{-15}
ZIK1	284307	19q13.43	5.28	6.37	1.16	0.84	−1.09	2.91 x 10^{-16}	2.01 x 10^{-15}
GOLGA8B	440270	15q14	9.53	7.92	1.65	1.71	1.61	3.20 x 10^{-16}	2.19 x 10^{-15}
ZKSCAN8	7745	6p22.1	7.48	8.9	1.54	0.59	−1.41	3.25 x 10^{-16}	2.22 x 10^{-15}
INPP4B	8821	4q31.21	7.08	8.19	1.18	0.97	−1.12	3.39 x 10^{-16}	2.31 x 10^{-15}
MST1	4485	3p21.31	9.39	8.15	1.28	1.3	1.24	3.62 x 10^{-16}	2.46 x 10^{-15}
F13A1	2162	6p25.1	8.62	10.14	1.57	1.56	−1.51	4.21 x 10^{-16}	2.84 x 10^{-15}
MPEG1	219972	11q12.1	9.46	10.42	1.01	0.99	−0.97	4.31 x 10^{-16}	2.91 x 10^{-15}
MSR1	4481	8p22	9.18	10.23	1.09	1.13	−1.05	4.36 x 10^{-16}	2.94 x 10^{-15}
CALCRL	10203	2q32.1	9.98	11.18	1.28	1	−1.2	4.42 x 10^{-16}	2.98 x 10^{-15}
ASMTL−AS1	80161	Xp22.33 and Yp11.2	7.03	5.15	1.94	2	1.88	4.57 x 10^{-16}	3.07 x 10^{-15}
TLCD1	116238	17q11.2	6.38	5.29	1.13	1.17	1.09	4.58 x 10^{-16}	3.07 x 10^{-15}
FAHD2CP	729234	2q11.2	5.23	4.19	1.11	0.89	1.04	4.63 x 10^{-16}	3.10 x 10^{-15}
ROBO1	6091	3p12.3	7.99	9.12	1.21	0.96	−1.13	4.77 x 10^{-16}	3.19 x 10^{-15}
COL15A1	1306	9q22.33	10.13	11.32	1.24	1.16	−1.18	4.94 x 10^{-16}	3.30 x 10^{-15}
FGL2	10875	7q11.23	10	11.03	1.1	0.92	−1.03	5.41 x 10^{-16}	3.60 x 10^{-15}
TXLNG	55787	Xp22.2	6.26	7.42	1.28	0.61	−1.16	5.47 x 10^{-16}	3.64 x 10^{-15}
PTPRB	5787	12q15	10.37	11.58	1.28	1.17	−1.22	5.65 x 10^{-16}	3.75 x 10^{-15}
CCNL2	81669	1p36.33	11.39	10.21	1.25	1.17	1.18	6.45 x 10^{-16}	4.26 x 10^{-15}
TLR1	7096	4p14	7.14	8.14	1.07	0.87	−1	8.48 x 10^{-16}	5.52 x 10^{-15}
LOC401052	401052	3p25.3	4.66	3.65	1.08	0.96	1.01	9.78 x 10^{-16}	6.34 x 10^{-15}
THBS1	7057	15q14	12.26	13.38	1.22	0.88	−1.11	1.12 x 10^{-15}	7.19 x 10^{-15}
LINC00174	285908	7q11.21	8.05	6.88	1.26	1.11	1.18	1.13 x 10^{-15}	7.22 x 10^{-15}
LPIN3	64900	20q12	10.38	9.35	1.11	0.92	1.03	1.27 x 10^{-15}	8.08 x 10^{-15}
PILRB	29990	7q22.1	10.82	9.48	1.42	1.36	1.33	1.30 x 10^{-15}	8.25 x 10^{-15}
ADAMTS12	81792	5p13.3–p13.2	3.77	5.47	1.82	1.7	−1.7	1.31 x 10^{-15}	8.30 x 10^{-15}
TNIK	23043	3q26.2–q26.31	7.56	8.66	1.21	0.79	−1.1	1.45 x 10^{-15}	9.12 x 10^{-15}

ZNF486	90649	19p12	5.35	6.57	1.33	1.04	−1.22	1.73 x 10^{-15}	1.08 x 10^{-14}
CSAD	51380	12q13.13	8.91	7.73	1.28	1.16	1.18	1.80 x 10^{-15}	1.12 x 10^{-14}
GTF2I	2969	7q11.23	10.36	11.33	1.09	0.54	−0.97	1.90 x 10^{-15}	1.17 x 10^{-14}
TMEM160	54958	19q13.32	6.81	5.78	1.11	1.03	1.02	2.36 x 10^{-15}	1.44 x 10^{-14}
F2R	2149	5q13.3	10.39	11.45	1.17	0.91	−1.06	2.45 x 10^{-15}	1.50 x 10^{-14}
EPAS1	2034	2p21	13.17	14.11	1.03	0.89	−0.95	2.50 x 10^{-15}	1.52 x 10^{-14}
PLA2G4A	5321	1q31.1	5.59	6.68	1.19	0.98	−1.09	2.58 x 10^{-15}	1.57 x 10^{-14}
ITGB3	3690	17q21.32	7.95	9.22	1.43	0.94	−1.28	2.62 x 10^{-15}	1.59 x 10^{-14}
HGF	3082	7q21.11	5.8	7.33	1.68	1.35	−1.53	2.77 x 10^{-15}	1.68 x 10^{-14}
FNIP2	57600	4q32.1	9.1	10.23	1.26	0.81	−1.13	2.95 x 10^{-15}	1.78 x 10^{-14}
DHX33	56919	17p13.2	7.18	8.18	1.14	0.45	−1	3.16 x 10^{-15}	1.90 x 10^{-14}
LRCH3	84859	3q29	7.19	8.15	1.1	0.44	−0.96	3.71 x 10^{-15}	2.22 x 10^{-14}
GJA1	2697	6q22.31	10.73	11.84	1.25	0.95	−1.11	5.96 x 10^{-15}	3.47 x 10^{-14}
FAM131C	348487	1p36.13	7.49	6.21	1.37	1.57	1.28	6.72 x 10^{-15}	3.89 x 10^{-14}
FAM160A1	729830	4q31.3	5.16	6.25	1.21	1.09	−1.09	7.48 x 10^{-15}	4.30 x 10^{-14}
TNFRSF14−AS 1	115110	1p36.32	8.52	7.54	1.05	1.17	0.97	8.14 x 10^{-15}	4.67 x 10^{-14}
RNFT2	84900	12q24.22	5.79	4.83	1.07	0.9	0.96	8.38 x 10^{-15}	4.80 x 10^{-14}
TM7SF2	7108	11q13.1	8.93	7.95	1.09	0.97	0.98	8.58 x 10^{-15}	4.90 x 10^{-14}
FGD5	152273	3p25.1	9.59	10.53	1.05	1.02	−0.95	1.03 x 10^{-14}	5.81 x 10^{-14}
TLR3	7098	4q35.1	9.29	10.53	1.4	1.1	−1.24	1.03 x 10^{-14}	5.82 x 10^{-14}
SLC27A5	10998	19q13.43	6.68	5.68	1.15	0.7	1	1.05 x 10^{-14}	5.93 x 10^{-14}
GPR34	2857	Xp11.4	7.28	8.29	1.11	1.15	−1.01	1.05 x 10^{-14}	5.95 x 10^{-14}
RSRP1	57035	1p36.11	9.4	8.43	1.05	1.16	0.97	1.14 x 10^{-14}	6.42 x 10^{-14}
SEC31B	25956	10q24.31	7.61	6.31	1.4	1.65	1.3	1.23 x 10^{-14}	6.90 x 10^{-14}
SGPP2	130367	2q36.1	6.96	8.17	1.36	1.16	−1.21	1.32 x 10^{-14}	7.39 x 10^{-14}
MAGIX	79917	Xp11.23	7.26	6.16	1.24	1.11	1.1	2.01 x 10^{-14}	1.10 x 10^{-13}
KCNN3	3782	1q21.3	5.92	7	1.21	1.19	−1.08	2.40 x 10^{-14}	1.31 x 10^{-13}
FAT4	79633	4q28.1	8.58	9.58	1.15	0.93	−1	2.50 x 10^{-14}	1.36 x 10^{-13}
DUSP23	54935	1q23.2	10.7	9.75	1.1	0.82	0.95	2.80 x 10^{-14}	1.51 x 10^{-13}
COL8A1	1295	3q12.1	8.09	9.61	1.78	1.2	−1.52	2.89 x 10^{-14}	1.56 x 10^{-13}
KCNJ5	3762	11q24.3	5.23	6.55	1.51	1.38	−1.33	3.06 x 10^{-14}	1.64 x 10^{-13}
ATXN7L2	127002	1p13.3	7.01	6.05	1.1	0.88	0.95	3.15 x 10^{-14}	1.69 x 10^{-13}
THSD7A	221981	7p21.3	6.44	7.88	1.69	1.19	−1.44	3.38 x 10^{-14}	1.81 x 10^{-13}
IL1RAP	3556	3q28	7.51	8.51	1.17	0.82	−1	3.46 x 10^{-14}	1.84 x 10^{-13}
CR1	1378	1q32.2	4.57	5.93	1.51	1.66	−1.36	3.85 x 10^{-14}	2.04 x 10^{-13}
GOLGA6L9	440295	15q25.2	7.22	6.14	1.24	1.14	1.08	3.95 x 10^{-14}	2.09 x 10^{-13}
RNF208	727800	9q34.3	7.32	6.32	1.15	1	1	4.97 x 10^{-14}	2.60 x 10^{-13}
KIF2A	3796	5q12.1	8.04	9.02	1.16	0.87	−0.99	5.17 x 10^{-14}	2.70 x 10^{-13}
MROH7	374977	1p32.3	6.56	5.07	1.7	1.7	1.5	5.27 x 10^{-14}	2.75 x 10^{-13}
EFNB2	1948	13q33.3	10.09	11.09	1.17	0.91	−1	5.49 x 10^{-14}	2.86 x 10^{-13}
PIEZO2	63895	18p11.22−p11.21	7.3	8.62	1.55	1.24	−1.32	6.42 x 10^{-14}	3.33 x 10^{-13}
ADGRF5	221395	6p12.3	11.18	12.13	1.09	1.01	−0.95	6.92 x 10^{-14}	3.57 x 10^{-13}
NRBP2	340371	8q24.3	10.59	9.62	1.15	0.88	0.97	7.31 x 10^{-14}	3.75 x 10^{-13}
KDR	3791	4q12	11.06	12.29	1.44	1.23	−1.23	7.59 x 10^{-14}	3.89 x 10^{-13}
PTPRC	5788	1q31.3−q32.1	9.67	10.68	1.18	1.05	−1.01	7.75 x 10^{-14}	3.96 x 10^{-13}
MZT2A	653784	2q21.1	9.94	8.93	1.18	1.02	1.01	7.82 x 10^{-14}	4.00 x 10^{-13}
ZNF827	152485	4q31.21−q31.22	6.84	7.82	1.2	0.61	−0.99	7.90 x 10^{-14}	4.03 x 10^{-13}
CAPN3	825	15q15.1	8.6	7.27	1.55	1.38	1.33	8.14 x 10^{-14}	4.15 x 10^{-13}
DOCK2	1794	5q35.1	8.4	9.43	1.18	1.16	−1.02	8.20 x 10^{-14}	4.18 x 10^{-13}
PBX1	5087	1q23.3	9.02	10.02	1.16	1.05	−1	9.90 x 10^{-14}	5.01 x 10^{-13}
SDHAP3	728609	5p15.33	9.18	8.23	1.13	0.96	0.95	1.36 x 10^{-13}	6.78 x 10^{-13}
MAN1A1	4121	6q22.31	9.55	10.57	1.25	0.8	−1.03	1.52 x 10^{-13}	7.55 x 10^{-13}
TMEM178A	130733	2p22.1	5.91	4.69	1.41	1.45	1.22	1.58 x 10^{-13}	7.85 x 10^{-13}
RPL13AP3	645683	14q22.3	4.03	3.08	1.12	1.02	0.95	2.09 x 10^{-13}	1.02 x 10^{-12}
ZNF462	58499	9q31.2	8.05	9.04	1.22	0.74	−0.99	2.42 x 10^{-13}	1.18 x 10^{-12}
CCN4	8840	8q24.22	6.19	7.24	1.25	1.18	−1.05	2.80 x 10^{-13}	1.35 x 10^{-12}
S1PR1	1901	1p21.2	9.95	10.99	1.27	1.01	−1.04	3.02 x 10^{-13}	1.45 x 10^{-12}
OVGP1	5016	1p13.2	6.36	5.17	1.47	0.99	1.19	3.19 x 10^{-13}	1.52 x 10^{-12}
SNHG3	8420	1p35.3	5.7	4.62	1.3	1.19	1.09	3.36 x 10^{-13}	1.60 x 10^{-12}
ALDH6A1	4329	14q24.3	7.64	8.89	1.54	1.13	−1.25	3.52 x 10^{-13}	1.68 x 10^{-12}
CPT1B	1375	22q13.33	8.48	7.1	1.64	1.63	1.38	3.60 x 10^{-13}	1.71 x 10^{-12}
SYNPO2	171024	4q26	9.34	10.42	1.27	1.32	−1.08	3.94 x 10^{-13}	1.86 x 10^{-12}
GPC6	10082	13q31.3−q32.1	9.2	10.38	1.46	1.08	−1.18	4.04 x 10^{-13}	1.91 x 10^{-12}
PABPC1L	80336	20q13.12	8.68	7.15	1.85	1.66	1.53	4.46 x 10^{-13}	2.10 x 10^{-12}
FPR1	2357	19q13.41	6.71	7.86	1.39	1.27	−1.16	4.55 x 10^{-13}	2.14 x 10^{-12}
ST6GALNAC 3	256435	1p31.1	6.5	7.59	1.35	1	−1.09	4.85 x 10^{-13}	2.27 x 10^{-12}
FAM124B	79843	2q36.2	4.65	5.65	1.2	1.21	−1.01	4.87 x 10^{-13}	2.28 x 10^{-12}
LCAT	3931	16q22.1	7.75	6.69	1.29	1.1	1.06	5.23 x 10^{-13}	2.44 x 10^{-12}
RPPH1	85495	14q11.2	5.87	4.4	1.79	1.57	1.47	6.77 x 10^{-13}	3.12 x 10^{-12}

SULF1	23213	8q13.2–q13.3	10.02	11.13	1.36	1.19	−1.1	8.55×10^{-13}	3.91×10^{-12}
SOCS1	8651	16p13.13	7.48	6.41	1.31	1.2	1.07	9.89×10^{-13}	4.49×10^{-12}
RUSC1−AS1	284618	1q22	6.37	5.42	1.16	1.2	0.96	1.27×10^{-12}	5.70×10^{-12}
EIF4EBP1	1978	8p11.23	10.27	9.33	1.18	1.03	0.95	1.29×10^{-12}	5.79×10^{-12}
GUSBP11	91316	22q11.23	9.24	8.21	1.23	1.42	1.03	1.45×10^{-12}	6.45×10^{-12}
ST8SIA4	7903	5q21.1	9.23	10.23	1.24	1.05	−1	1.49×10^{-12}	6.64×10^{-12}
GAMT	2593	19p13.3	10.6	9.52	1.34	1.17	1.07	1.57×10^{-12}	6.99×10^{-12}
FLT1	2321	13q12.3	12.29	13.41	1.41	1.13	−1.12	1.63×10^{-12}	7.22×10^{-12}
PNPLA7	375775	9q34.3	8.63	7.53	1.34	1.35	1.1	1.66×10^{-12}	7.36×10^{-12}
ADAMTSL3	57188	15q25.2	7.66	9	1.69	1.41	−1.35	1.69×10^{-12}	7.47×10^{-12}
VWF	7450	12p13.31	13.33	14.41	1.35	1.24	−1.08	2.09×10^{-12}	9.16×10^{-12}
RNF207	388591	1p36.31	8.16	7.06	1.38	1.25	1.1	2.18×10^{-12}	9.54×10^{-12}
CHCHD10	400916	22q11.23	10.26	9.28	1.24	1	0.98	2.19×10^{-12}	9.55×10^{-12}
SYDE2	84144	1p22.3	5.48	6.44	1.23	0.9	−0.96	2.34×10^{-12}	1.02×10^{-11}
MIR503HG	84848	Xq26.3	5.92	4.74	1.49	1.32	1.19	2.66×10^{-12}	1.15×10^{-11}
WDFY3−AS2	404201	4q21.23	5.53	6.58	1.36	0.92	−1.05	2.70×10^{-12}	1.17×10^{-11}
PABPC4L	132430	4q28.3	6.65	7.6	1.21	0.99	−0.95	2.91×10^{-12}	1.25×10^{-11}
FLRT2	23768	14q31.3	5.29	6.41	1.38	1.46	−1.12	3.10×10^{-12}	1.33×10^{-11}
AHSA2P	130872	2p15	9.6	8.52	1.36	1.3	1.08	3.91×10^{-12}	1.66×10^{-11}
TTLL3	26140	3p25.3	9.59	8.58	1.28	1.2	1.01	4.03×10^{-12}	1.71×10^{-11}
AOAH	313	7p14.2	7.46	8.44	1.24	1.16	−0.98	4.08×10^{-12}	1.73×10^{-11}
CFAP70	118491	10q22.2	6.8	5.81	1.23	1.28	0.99	4.12×10^{-12}	1.75×10^{-11}
BICD1	636	12p11.21	7.37	8.36	1.3	0.83	−0.98	5.24×10^{-12}	2.19×10^{-11}
GOLGA8A	23015	15q14	10.34	9.01	1.68	1.7	1.33	5.60×10^{-12}	2.34×10^{-11}
MAPK8IP3	23162	16p13.3	10.82	9.82	1.29	1.2	1.01	8.52×10^{-12}	3.50×10^{-11}
AGER	177	6p21.32	6.87	5.68	1.54	1.39	1.19	9.17×10^{-12}	3.76×10^{-11}
EPHA4	2043	2q36.1	7.61	8.59	1.28	1.07	−0.98	9.30×10^{-12}	3.81×10^{-11}
MYSM1	114803	1p32.1	5.99	6.97	1.32	0.8	−0.98	9.40×10^{-12}	3.84×10^{-11}
CLIC5	53405	6p21.1	6.62	7.81	1.52	1.5	−1.19	9.69×10^{-12}	3.96×10^{-11}
ARHGAP33	115703	19q13.12	8.16	7.03	1.47	1.28	1.13	9.97×10^{-12}	4.06×10^{-11}
COL12A1	1303	6q13–q14.1	9.54	10.52	1.27	1.2	−0.98	1.09×10^{-11}	4.44×10^{-11}
RARRES2	5919	7q36.1	12.68	11.64	1.32	1.38	1.04	1.11×10^{-11}	4.50×10^{-11}
TMEM150C	441027	4q21.22	7.49	8.63	1.51	1.14	−1.14	1.15×10^{-11}	4.68×10^{-11}
AMY2B	280	1p21.1	8.67	7.37	1.7	1.48	1.3	1.34×10^{-11}	5.40×10^{-11}
GNRH1	2796	8p21.2	5.96	4.79	1.54	1.37	1.17	1.65×10^{-11}	6.61×10^{-11}
TMEM74B	55321	20p13	7.28	6.2	1.46	1.16	1.09	2.04×10^{-11}	8.10×10^{-11}
PCSK5	5125	9q21.13	6.27	7.34	1.46	1	−1.07	2.46×10^{-11}	9.67×10^{-11}
MST1P2	11209	1p36.13	7.46	6.15	1.69	1.84	1.31	2.59×10^{-11}	1.01×10^{-10}
APOLD1	81575	12p13.1	11.1	12.18	1.41	1.41	−1.08	2.61×10^{-11}	1.02×10^{-10}
HIST1H1C	3006	6p22.2	10.7	9.66	1.39	1.27	1.05	3.13×10^{-11}	1.22×10^{-10}
TSPAN5	10098	4q23	4.74	6.01	1.75	1.17	−1.27	3.42×10^{-11}	1.33×10^{-10}
TEKT4P2	100132288	21p11.2	8.28	7.33	1.26	1.24	0.95	3.59×10^{-11}	1.38×10^{-10}
SIGLEC5	8778	19q13.41	4.29	5.32	1.39	1.18	−1.03	3.56×10^{-11}	1.38×10^{-10}
CD209	30835	19p13.2	6	7.14	1.54	1.3	−1.13	3.98×10^{-11}	1.53×10^{-10}
RASGRF2	5924	5q14.1	7.21	8.17	1.3	1.11	−0.96	4.10×10^{-11}	1.57×10^{-10}
DNAH17	8632	17q25.3	5.32	4.35	1.33	1.02	0.97	4.22×10^{-11}	1.62×10^{-10}
PXK	54899	3p14.3	6.55	7.53	1.37	0.78	−0.97	4.68×10^{-11}	1.78×10^{-10}
LOC155060	155060	7q36.1	7.86	6.77	1.49	1.28	1.09	5.01×10^{-11}	1.90×10^{-10}
CXADR	1525	21q21.1	5.86	7.23	1.85	1.7	−1.37	5.03×10^{-11}	1.91×10^{-10}
SULT1C2	6819	2q12.3	9.75	8.66	1.45	1.51	1.09	5.38×10^{-11}	2.03×10^{-10}
PRSS53	339105	16p11.2	6.51	5.43	1.5	1.09	1.08	5.62×10^{-11}	2.12×10^{-10}
CASS4	57091	20q13.31	5.15	6.12	1.33	1.17	−0.98	6.18×10^{-11}	2.31×10^{-10}
LINC00893	100131434	Xq28	5.9	4.69	1.62	1.66	1.22	6.89×10^{-11}	2.56×10^{-10}
CAPN12	147968	19q13.2	10.32	9.05	1.78	1.3	1.28	7.30×10^{-11}	2.71×10^{-10}
SLC1A3	6507	5p13.2	7.66	8.68	1.4	1.21	−1.01	8.54×10^{-11}	3.14×10^{-10}
LGI2	55203	4p15.2	5.16	6.11	1.33	1.22	−0.96	1.65×10^{-10}	5.90×10^{-10}
PCDHGA9	56107	5q31.3	5.12	6.62	2.12	1.74	−1.5	1.68×10^{-10}	6.01×10^{-10}
TNC	3371	9q33.1	9.87	11.08	1.77	1.42	−1.21	5.00×10^{-10}	1.70×10^{-9}
FKBP9P1	360132	7p11.2	5.55	6.54	1.43	1.27	−0.99	5.30×10^{-10}	1.80×10^{-9}
CDH13	1012	16q23.3	9.76	10.71	1.4	1.07	−0.95	6.06×10^{-10}	2.05×10^{-9}
GALNT15	117248	3p25.1	7.93	9.02	1.56	1.5	−1.09	6.25×10^{-10}	2.11×10^{-9}
SLC16A7	9194	12q14.1	4.54	5.77	1.82	1.39	−1.23	7.13×10^{-10}	2.39×10^{-9}
TNFRSF25	8718	1p36.31	6.75	5.62	1.64	1.49	1.13	7.64×10^{-10}	2.55×10^{-9}
SEMA3A	10371	7q21.11	3.72	4.89	1.68	1.69	−1.17	7.70×10^{-10}	2.57×10^{-9}
CELF6	60677	15q23	6.94	5.86	1.56	1.46	1.08	7.85×10^{-10}	2.62×10^{-9}
PLA2R1	22925	2q24.2	6.28	7.35	1.61	1.08	−1.07	7.98×10^{-10}	2.66×10^{-9}
APLNR	187	11q12.1	9.36	10.32	1.37	1.4	−0.96	8.43×10^{-10}	2.80×10^{-9}
HSF4	3299	16q22.1	10.25	8.94	1.92	1.66	1.31	8.51×10^{-10}	2.83×10^{-9}
PGAM2	5224	7p13	5.85	4.74	1.59	1.56	1.11	8.80×10^{-10}	2.92×10^{-9}
HRH2	3274	5q35.2	6.19	7.63	2.13	1.77	−1.44	9.40×10^{-10}	3.11×10^{-9}
EPHA3	2042	3p11.1	7.32	8.32	1.45	1.42	−1	1.01×10^{-9}	3.33×10^{-9}

MLXIPL	51085	7q11.23	9.29	8.02	1.81	1.89	1.26	1.02 x 10^{-9}	3.38 x 10^{-9}
EDNRB	1910	13q22.3	10.66	11.71	1.57	1.29	−1.05	1.30 x 10^{-9}	4.26 x 10^{-9}
DNAAF3	352909	19q13.42	4.8	3.7	1.6	1.62	1.1	1.36 x 10^{-9}	4.42 x 10^{-9}
LDLR	3949	19p13.2	7.25	8.2	1.42	1.22	−0.95	1.51 x 10^{-9}	4.90 x 10^{-9}
FHL5	9457	6q16.1	6.86	7.91	1.55	1.47	−1.05	1.69 x 10^{-9}	5.43 x 10^{-9}
AKR7L	246181	1p36.13|1p35–p36.1	6.42	5.39	1.55	1.39	1.03	2.22 x 10^{-9}	7.09 x 10^{-9}
CX3CR1	1524	3p22.2	7.61	8.56	1.4	1.51	−0.96	2.45 x 10^{-9}	7.77 x 10^{-9}
MYH7B	57644	20q11.22	4.94	3.84	1.67	1.45	1.1	2.54 x 10^{-9}	8.03 x 10^{-9}
PDGFD	80310	11q22.3	9.89	10.84	1.48	1.09	−0.95	2.79 x 10^{-9}	8.78 x 10^{-9}
LYZ	4069	12q15	11.17	12.21	1.59	1.35	−1.03	3.82 x 10^{-9}	1.19 x 10^{-8}
AKAP12	9590	6q25.1	11.04	11.99	1.49	1.12	−0.95	3.96 x 10^{-9}	1.23 x 10^{-8}
MAMDC2	256691	9q21.12	4.03	5.03	1.49	1.59	−1	4.22 x 10^{-9}	1.30 x 10^{-8}
A1BG	1	19q13.43	6.09	5.12	1.54	1.05	0.97	5.03 x 10^{-9}	1.54 x 10^{-8}
GOLGA2P5	55592	12q23.1	8.14	7.19	1.5	1.27	0.95	7.30 x 10^{-9}	2.20 x 10^{-8}
PLIN5	440503	19p13.3	5.42	4.17	1.96	1.73	1.25	7.37 x 10^{-9}	2.22 x 10^{-8}
TLL1	7092	4q32.3	7.03	8.21	1.91	1.35	−1.18	8.38 x 10^{-9}	2.50 x 10^{-8}
PLCB1	23236	20p12.3	9.04	10.04	1.63	1.08	−1	1.14 x 10^{-8}	3.37 x 10^{-8}
CHST13	166012	3q21.3	8.09	7.02	1.65	1.71	1.06	1.38 x 10^{-8}	4.01 x 10^{-8}
COL14A1	7373	8q24.12	8.57	9.72	1.84	1.6	−1.15	1.55 x 10^{-8}	4.51 x 10^{-8}
KCNIP3	30818	2q11.1	7.99	6.77	1.97	1.71	1.22	1.64 x 10^{-8}	4.75 x 10^{-8}
NCR3LG1	374383	11p15.1	6.51	7.48	1.57	1.35	−0.97	1.82 x 10^{-8}	5.24 x 10^{-8}
HERC2P2	400322	15q11.2	9.67	8.7	1.57	1.45	0.97	2.52 x 10^{-8}	7.14 x 10^{-8}
SELP	6403	1q24.2	6.48	7.51	1.72	1.33	−1.03	3.25 x 10^{-8}	9.08 x 10^{-8}
TMEM266	123591	15q24.2	5.38	4.29	1.78	1.72	1.09	4.29 x 10^{-8}	1.19 x 10^{-7}
PCDHB3	56132	5q31.3	5.37	6.38	1.68	1.48	−1.01	4.38 x 10^{-8}	1.21 x 10^{-7}
PCDH7	5099	4p15.1	5.04	6.1	1.73	1.76	−1.06	5.12 x 10^{-8}	1.40 x 10^{-7}
AP1M2	10053	19p13.2	8.07	7.05	1.71	1.65	1.02	7.94 x 10^{-8}	2.13 x 10^{-7}
C16ORF74	404550	16q24.1	7.17	5.92	2.15	1.82	1.26	8.66 x 10^{-8}	2.31 x 10^{-7}
POSTN	10631	13q13.3	9.62	10.72	1.89	1.62	−1.1	8.96 x 10^{-8}	2.39 x 10^{-7}
DNASE1	1773	16p13.3	5.94	4.92	1.77	1.3	1.01	9.05 x 10^{-8}	2.41 x 10^{-7}
PGGHG	80162	11p15.5	10.97	9.74	2.16	1.7	1.22	1.57 x 10^{-7}	4.10 x 10^{-7}
FER1L4	80307	20q11.22	8.65	7.22	2.57	2	1.43	2.38 x 10^{-7}	6.07 x 10^{-7}
KLHL4	56062	Xq21.31	4.8	5.94	2.05	1.8	−1.14	3.47 x 10^{-7}	8.68 x 10^{-7}
PLCXD3	345557	5p13.1	4.7	5.85	2.07	1.79	−1.15	3.54 x 10^{-7}	8.87 x 10^{-7}
ADCY10P1	221442	6p21.1	5.61	4.65	1.69	1.7	0.95	4.17 x 10^{-7}	1.04 x 10^{-6}
CARD14	79092	17q25.3	5.61	4.48	2.05	1.81	1.13	4.93 x 10^{-7}	1.21 x 10^{-6}
PDGFRA	5156	4q12	6.06	7.26	2.1	2.36	−1.2	5.13 x 10^{-7}	1.26 x 10^{-6}
VGLL3	389136	3p12.1	5.61	6.58	1.73	1.74	−0.97	5.26 x 10^{-7}	1.29 x 10^{-6}
ITGA11	22801	15q23	7.14	8.1	1.76	1.6	−0.96	6.30 x 10^{-7}	1.53 x 10^{-6}
GPD1	2819	12q13.12	10.02	8.91	1.98	2.18	1.11	8.54 x 10^{-7}	2.05 x 10^{-6}
LOC100130872	100130872	4p16.3	6.48	5.51	1.88	1.49	0.97	1.41 x 10^{-6}	3.30 x 10^{-6}
LAMA2	3908	6q22.33	5.75	6.69	1.73	1.92	−0.95	1.42 x 10^{-6}	3.31 x 10^{-6}
GPT	2875	8q24.3	8.25	7.25	1.83	1.94	0.99	1.43 x 10^{-6}	3.34 x 10^{-6}
PREX2	80243	8q13.2	6.88	8.2	2.66	1.64	−1.32	1.95 x 10^{-6}	4.49 x 10^{-6}
NTRK2	4915	9q21.33	7.72	8.79	2.14	1.83	−1.06	3.90 x 10^{-6}	8.70 x 10^{-6}
VCAN	1462	5q14.2–q14.3	11.51	12.48	1.97	1.54	−0.97	4.00 x 10^{-6}	8.92 x 10^{-6}
BAIAP2L2	80115	22q13.1	9.01	8	2.02	1.8	1.01	4.17 x 10^{-6}	9.27 x 10^{-6}
DOC2A	8448	16p11.2	8.75	7.43	2.71	2.23	1.33	5.05 x 10^{-6}	1.11 x 10^{-5}
MMRN1	22915	4q22.1	6.83	7.85	2.04	1.92	−1.02	5.13 x 10^{-6}	1.13 x 10^{-5}
C7	730	5p13.1	8.28	9.55	2.53	2.6	−1.27	6.46 x 10^{-6}	1.41 x 10^{-5}
SULT1C4	27233	2q12.3	7.32	8.53	2.56	1.9	−1.21	7.33 x 10^{-6}	1.59 x 10^{-5}
NPR3	4883	5p13.3	9.91	10.98	2.35	1.83	−1.07	1.87 x 10^{-5}	3.86 x 10^{-5}
ADGRG2	10149	Xp22.13	4.49	5.54	2.24	2.25	−1.05	2.20 x 10^{-5}	4.51 x 10^{-5}
DEFB1	1672	8p23.1	10.15	9.02	2.5	2.09	1.12	2.59 x 10^{-5}	5.28 x 10^{-5}

Note: DEGs are defined by q < 0.0001, fold change log2 Ratio ≥ |0.95|.

Table S2. Demographics of patient populations.

	Training	Column 1	Testing	Column 2
**Characteristics**	*n*	%	*n*	%
Fatality; NO	191	0.656357388	141	0.677884615
Fatality; YES	100	0.343642612	67	0.322115385
follow up (Months), median (Q1–Q3)	43.5 (18.5–66.1)		37.2 (16.2–61.6)	

Age at diagnosis (Year), median (Q1–Q3)	61.5 (52–70.3)		60 (51–69)	
Female	103	0.343333333	80	0.384615385
Male	197	0.656666667	128	0.615384615
Stage I	145	0.483333333	102	0.490384615
Stage II	25	0.083333333	29	0.139423077
Stage III	75	0.25	49	0.235576923
Stage IV	55	0.183333333	28	0.134615385
Total	300		208	
Grade G1	5	0.016666667	7	0.033653846
Grade G2	120	0.4	94	0.451923077
Grade G3	125	0.416666667	74	0.355769231
Grade G4	47	0.156666667	28	0.134615385
Grade Gx	3	0.01	2	0.009615385
M0	236	0.786666667	165	0.793269231
M1	51	0.17	27	0.129807692
MX	13	0.043333333	14	0.067307692
T1	150	0.5	102	0.490384615
T2	35	0.116666667	31	0.149038462
T3	109	0.363333333	70	0.336538462
T4	6	0.02	5	0.024038462

© 2020 by the authors. Licensee MDPI, Basel, Switzerland. This article is an open access article distributed under the terms and conditions of the Creative Commons Attribution (CC BY) license (http://creativecommons.org/licenses/by/4.0/).
